# Using Central IRBs for Multicenter Clinical Trials in the United States

**DOI:** 10.1371/journal.pone.0054999

**Published:** 2013-01-30

**Authors:** Kathryn E. Flynn, Cynthia L. Hahn, Judith M. Kramer, Devon K. Check, Carrie B. Dombeck, Soo Bang, Jane Perlmutter, Felix A. Khin-Maung-Gyi, Kevin P. Weinfurt

**Affiliations:** 1 Duke Clinical Research Institute, Duke University School of Medicine, Durham, North Carolina, United States of America; 2 Department of Psychiatry and Behavioral Sciences, Duke University School of Medicine, Durham, North Carolina, United States of America; 3 Office of Research Compliance, Feinstein Institute for Medical Research, North Shore-Long Island Health System, Manhasset, New York, United States of America; 4 Department of Medicine, Duke University School of Medicine, Durham, North Carolina, United States of America; 5 Celgene Corporation, Summit, New Jersey, United States of America; 6 Gemini Group, Ann Arbor, Michigan, United States of America; 7 Chesapeake IRB, Columbia, Maryland, United States of America; Glaxo Smith Kline, Denmark

## Abstract

Research institutions differ in their willingness to defer to a single, central institutional review board (IRB) for multicenter clinical trials, despite statements from the FDA, OHRP, and NIH in support of using central IRBs to improve the efficiency of conducting trials. The Clinical Trials Transformation Initiative (CTTI) supported this project to solicit current perceptions of barriers to the use of central IRBs and to formulate potential solutions. We held discussions with IRB experts, interviewed representatives of research institutions, and held an expert meeting with diverse stakeholder groups and thought leaders. We found that many perceived barriers relate to conflating responsibilities of the institution with the ethical review responsibilities of the IRB. We identified the need for concrete tools to help research institutions separate institutional responsibilities from ethical responsibilities required of the IRB. One such tool is a document we created that delineates these responsibilities and how they might be assigned to each entity, or, in some cases, both entities. This tool and project recommendations will be broadly disseminated to facilitate the use of central IRBs in multicenter trials. The ultimate goal is to increase the nation’s capacity to efficiently conduct the large number of high-quality trials.

## Introduction

Maximizing the efficiency of multicenter clinical trials so they can provide high-quality evidence to answer important medical questions is an important public health interest. As multicenter clinical trials have become more common, researchers have begun to question whether the goal of protecting research participants is enhanced by having each site’s local institutional review board (IRB) conduct a full review of multicenter protocols, which can add significant delays to study start-up [1], [2]. In addition, multiple reviews may result in differences in the way patients are treated at different sites (eg, because of differences in informed consent forms) for which there is no ethical justification but may cause confusion among participants.

To improve the efficiency of conducting multicenter clinical trials in the United States, the Food and Drug Administration (FDA), the Office of Human Research Protections (OHRP), and the Department of Health and Human Services (DHHS) support the use of central IRBs [3]–[5]. In July 2011, the DHHS invited commentary on their proposal to change the Common Rule to include mandated centralized review for multicenter trials [3]. Despite this support, research institutions differ in their willingness to defer to centralized IRB review [6], [7].

To facilitate the ethical and efficient conduct of multicenter trials, we sought to determine the barriers to using central IRBs for multicenter clinical trials in the United States, formulate solutions to overcome these barriers, obtain feedback on the proposed solutions from stakeholders at diverse US research institutions, and develop recommendations for implementing these solutions.

## Methods

The Clinical Trials Transformation Initiative (CTTI) supported this project to solicit current perceptions of barriers to the use of central IRBs and to formulate potential solutions. We conducted a review of the literature and held a series of discussions with 43 experts in the field–including representatives from institutional IRBs, federal IRBs, commercial IRBs, industry, and regulatory agencies–to arrive at an understanding of the barriers to central review and to generate solutions. We identified 33 published reports (unpublished data). Identified barriers included apprehension about regulatory and legal liability, issues regarding the local context of research, and logistic barriers such as loss of income from industry fees for IRB review. Table 1 summarizes the barriers and proposed solutions. Next, we conducted interviews with 25 stakeholders at 6 research institutions that do not routinely use central IRBs to obtain feedback on the proposed solutions. The research institutions had their own IRBs and were purposely selected to be diverse with respect to their volume of federally and industry-funded clinical research, the type of institution (eg, academic medical center or independent hospital), and geographic location. The stakeholders at each institution included an IRB chair, IRB administrator or manager, institutional general counsel, vice dean for research, director of clinical trials, or other individuals responsible for making decisions regarding the use of outside IRBs. Our goal was to identify the range of perceptions and beliefs among diverse participants, and not to establish the prevalence of different views. We obtained verbal consent to record these interviews, which we documented in the recordings and our notes. An author who did not participate in the interviews and did not know the names of the respondents performed analyses anonymously.

**Table 1:** Perceived Barriers to Using Centralized IRB Review in Multicenter Clinical Trials in the United States and Proposed Solutions.

Barrier	Potential Solutions
Feasibility of working with multiple outside IRBs, each requiring differentforms and/or electronic systems to submit a protocol	Identify standard data elements to facilitate review and reporting across disparate systems.
Loss of revenue generated from fees for institutional IRB review of studieswith commercial sponsors	Charge an administrative fee for institutional responsibilities. (Institutions may need to find a new way to cover fixed costs for the IRB for non-sponsored activities.)
Concern about regulatory liability in the event of noncompliance	Clarify OHRP policy to take action against the IRB of record as opposed to participating sites for noncompliance with regulations.
Concern about legal liability in the event of litigation secondary to errors, omissions, or negligence of an IRB not directly affiliated with the institution conducting research	Establish liability protections through a well-defined communication plan and standard contracts with the outside IRB.
Quality of review, such as missing important human subject protectionsissues without redundant review, caliber/expertise of reviewers,and insufficient time spent on protocols	Conduct standardized tests of IRBs to demonstrate quality (eg, send a standardized protocol to an outside IRB and the local IRB to compare results). (Evaluating review quality is hampered without an agreed way to measure it.)
Potential loss of local context	In a well-defined relationship, the local institution retains authority to decide whether to participate in a study or to limit an investigator’s involvement. Consent forms can have a core that is the same for all sites, and a section customizable to the institution that addresses relevant state laws or institutional concerns regarding (eg, compensation for research-related injury, institutional contact information, surrogate consent, and costs of participation).

Abbreviations: IRB, institutional review board; OHRP, Office of Human Research Protections.

To further refine the solutions, analyze stakeholder reactions to the solutions, and develop policy recommendations, we subsequently convened a 2-day expert meeting in April 2012 with 47 representatives from a broad cross-section of the clinical research enterprise, including government and industry sponsors of clinical research, FDA, OHRP, academic and non-academic research institutions, commercial IRBs, and patient advocacy groups. A list of attendees is available on the CTTI website (https://www.ctti-clinicaltrials.org/website-administration/files/meeting%20attendees.pdf).

## Results and Discussion

### Need to Clarify Terms

Based on the initial expert discussions, an early finding was the need to clarify the term “central IRB.” Although many people refer to an independent or commercial IRB as a central IRB, an independent or commercial IRB can also be contracted by an institution to serve as the institution’s “local” IRB. In addition, the term “central IRB” is commonly used to describe other alternative models, such as facilitated, federated, and consortium models. In the federated and consortium models, each IRB in the group may agree upon reciprocal acceptance of one another’s IRB review and decision; however, this decision does not extend to IRBs outside of the group. In the facilitated model, the outside IRB and local IRBs share information; the outside IRB may review the protocol first and share its review with the IRBs at the local institutions to facilitate their reviews; however, this still involves review by multiple IRBs.

In the interviews with institutional stakeholders, we provided interviewees with a brief definition of central IRB, “a single IRB of record for a multicenter clinical trial,” and a longer definition, “a properly constituted IRB to which sites cede all regulatory responsibility for scientific oversight and integrity of the protocol from initial review to termination of the research, including review of informed consent.” These definitions were effective in clarifying the model of ethical review under consideration, suggesting that they might be useful in future policy discussions.

### Decoupling Institutional and Ethical Review Responsibilities

A major finding was that many of the perceived barriers to using central IRBs arise from the fact that most or all of the tasks related to protecting the institution (eg, conflict of interest review) are often coordinated through the institution’s IRB office and incorporated into their review process. What evolved as bureaucratic convenience in most institutions–locating certain institutional review processes in the IRB office–seems to have altered perceptions of what is entailed in the ethical review of research. This conflating of institutional responsibilities with the ethical review responsibilities of the IRB leads to confusion about how institutional responsibilities would be handled in the context of a central IRB review, creating resistance to using central IRBs.

To address this problem, we developed a guide for institutions that can help to decouple institutional and IRB responsibilities to assist in the acceptance of centralized ethical review, which has the potential to result in more consistent and efficient reviews. It outlines categories of legal and ethical responsibilities of an institution and an IRB in overseeing the conduct of clinical trials. Highlights of the guide are provided in **Table 2**; the detailed guide is provided in **Appendix S1**. This document is meant to support communication between institutions and central IRBs when assigning responsibilities for multicenter clinical trial protocols that are using a central IRB. Thus, it is most relevant for institutions that have their own local IRB. We solicited feedback on the guide during our interviews with institutional stakeholders and further refined it in the context of our expert meeting.

**Table 2:** Responsibilities of Institutions and Central IRBs for Multicenter Clinical Trial Protocols*.

Responsibility	Central IRB	Institution	Both	Either
Execute IRB authorization			×	
Assess investigator qualifications			×	
Research education and training of IRB personnel	×			
Register with FDA and OHRP	×			
Notify sites of accreditation changes	×			
Ensure ethical standards and regulations	×			
Collate site specific information	×			
Approve informed consent forms	×			
Provide copies of IRB decisions, rosters, & minutes	×			
Notify sites of non-compliance concerns	×			
Education and training of investigators and study coordinators		×		
Credentialing of staff		×		
Maintain FWAs		×		
Conduct security and privacy review for HIPAA		×		
Ensure investigator compliance and conflict of interest		×		
Evaluate local context				×
Provide waiver of consent if indicated				×

Abbreviations: IRB, institutional review board; FDA, Food and Drug Administration; FWA, federalwide assurance; HIPAA, Health Insurance Portability and Accountability Act; OHRP, Office of Human Research Protections.

* This table provides highlights of a guide for institutions that can help to decouple institutional and IRB responsibilities to assist in the acceptance of centralized ethical review; the detailed guide is provided in Appendix S1.

### Level of Comfort and Trust with Central IRB Review

The second major theme to emerge from our interviews was a feeling of discomfort with an external entity handling the ethical review and oversight of a multicenter protocol. Institutional stakeholders frequently made reference to issues of “comfort” and “trust” in the review by a central IRB. These issues appeared to be influenced by the institution’s previous experiences with outside IRBs. When an institution had no prior experience, there was less comfort and trust. Moreover, although IRB accreditation from the Association for the Accreditation of Human Research Protection Programs was important, it was not sufficient to alleviate these concerns. The data suggest that experience, not simply increased knowledge, is necessary to allow institutional stakeholders to feel more comfortable with central IRB review.

The majority of institutions have little motivation to participate in protocols with central IRB review, so gaining this experience may be difficult. Although industry sponsors would have an incentive to use a central IRB (believing it to be more efficient and less expensive), we could find no examples of industry sponsors who mandate use of a central IRB for all participating sites. In contrast, the National Institutes of Health (NIH) is in a unique position to make this happen, and there is now at least one example of a government sponsor, the National Institute of Neurological Disorders and Stroke NeuroNEXT Network [8], that has required the use of a central IRB. (This is in contrast to the National Cancer Institute’s CIRB, which is voluntary and functions more like a facilitated review.) Research institutions that want to participate in prestigious research networks like NeuroNEXT will likely accept review by a central IRB despite discomfort with the model because the use of the central IRB is a prerequisite to membership. Thus, we encourage the NIH and other government sponsors to consider requiring the use of central IRB review for some multisite trial networks so that relevant stakeholders can gain experience that will inform their levels of comfort and trust. Without such initiatives, there is little incentive for research sites to overcome their feelings of discomfort with central IRB review. Another advantage of providing more opportunities to participate in research networks with mandated central IRB review is that institutional stakeholders can observe how concerns about local context can be addressed.

### Addressing Concerns about Local Context

One of the most frequently cited barriers to using a central IRB was the idea that some aspects important to IRB review could not be adequately addressed by a central IRB, since an outside group may not have necessary knowledge about the site’s unique local context. Some specific examples we heard included local knowledge about investigators, the research setting, capacity to conduct the trial (resources), or unique patient populations. In some of these situations, institutional stakeholders were concerned about not wanting local knowledge to become public (eg, in the case of investigator conflicts of interest), while in other situations it was an issue of not having an opportunity to share the local information about unique populations. Many interviewees described a desire to protect their research subjects, whom they regarded as unique. As one interviewee commented, “these are our friends and family.” However, some people, including our own patient advocate, believe that there are few if any local differences that should result in patients being treated differently at different sites and that doing so may be unethical.

To address the concerns about local context, we considered feedback from our stakeholder interviews and the expert meeting. We recommend the following:

1. Clarify how and where local issues are reflected. In the detailed guide provided in Appendix S1, we outline the need for the institution and the central IRB to develop a detailed communication plan to share information about the site, the investigators, and other details of the trial. The guide also specifies that the central IRB should specify where local institutions should insert informed consent language specific to their state, for example, or the special populations they serve.
2. Reiterate the regulatory positions of OHRP and FDA. Both OHRP and FDA have published their support for using central IRBs for multicenter protocols [3]–[5]. Moreover, in the Advanced Notice of Proposed Rule Making (ANPRM), the DHHS position on local context reads, “Relevant local contextual issues (eg, investigator competence, site suitability) pertinent to most clinical studies can be addressed through mechanism other than local IRB review. For research where local perspectives might be distinctly important (eg, in relation to certain kinds of vulnerable populations targeted for recruitment) local IRB review could be limited to such consideration(s) but again, IRB review is not the only mechanism for addressing such issues. The evaluation of a study’s social value, scientific validity, and risks and benefits, and the adequacy of the informed consent document and process generally do not require the unique perspective of a local IRB” [3].

Yet, we still heard uneasiness from stakeholders on this topic. Dr. Jerry Menikoff, director of OHRP, recently published his perspective on the importance of standardization across sites [1], and he elaborated on the regulatory aspects of local context at the expert meeting. He brought clarity to the issue when he said, “OHRP clearly recognizes now that local context issues can be dealt with by an outside IRB…. If you put together the ANPRM quotes with the OHRP letter and FDA letters, you could hopefully convince institutions that you actually can do central review and certainly OHRP isn’t going to come after you for not dealing with local context appropriately.” These remarks are consistent with the idea that an outside, central IRB could reflect local context issues satisfactorily.

### Conclusions

We identified specific steps likely to facilitate adoption of central IRB review for multicenter clinical trials. The clinical trials community has an opportunity to significantly improve the quality and efficiency of one essential aspect of the clinical research enterprise, as there is good reason to believe that central IRB review would be beneficial to clinical research. The FDA, OHRP, and DHHS have already demonstrated their support for central IRB review. What is still needed is experience using the model under circumstances where there are potential solutions to anticipated barriers. We hope that the solutions proposed herein can maximize successful institutional collaboration with central IRBs to facilitate ethical and efficient conduct of multicenter trials.

## Supporting Information

Appendix S1 **Considerations to Support Communication Between Institutions and Outside IRBs When Responsibilities are Being Assigned for Multicenter Clinical Trial Protocols.**
(DOCX)
