# Supplementary material for: Using Central IRBs for Multicenter Clinical Trials in the United States
Source: PLoS One. 2013 Jan 30;8(1):e54999. doi: 10.1371/journal.pone.0054999 (PMC3559741; doi:10.1371/journal.pone.0054999)
Supplement: Appendix S1 — Considerations to Support Communication Between Institutions and Outside IRBs When Responsibilities are Being Assigned for Multicenter Clinical Trial Protocols. (DOCX) [file pone.0054999.s001.docx]

**Appendix S1.** Considerations to Support Communication Between Institutions and Outside IRBs When Responsibilities are Being Assigned for Multicenter Clinical Trial Protocols

The purpose of this document is to outline categories of legal and ethical responsibilities of an institution and an institutional review board (IRB) in overseeing the conduct of clinical trials. This document is meant to support communication between institutions and external central IRBs when responsibilities are being assigned for multicenter clinical trial protocols that are using a central IRB. This document is most relevant for institutions that have the option to use their own local IRB and should be used as a starting point for decoupling institutional and IRB responsibilities.

The **central IRB for a multicenter protocol** is the single IRB of record for the protocol. It has regulatory responsibility for assuring the protection of the rights and welfare of research participants from initial review to termination of the research, including review and approval of informed consent.

The **institution** is the local entity setting standards to determine whether a research investigator can conduct research under its auspices (e.g., allowing admitting privileges to a hospital, authorizing an investigator to use facilities to conduct research, or determining faculty status). Clinical sites participating in a multicenter protocol may, in some instances, not be associated with an institution. In these cases, the clinical investigator or the study sponsor would assume some of the institutional responsibilities.

1. Responsibilities that **both** the central IRB and the institution should assume:

1. Execute an IRB Authorization Agreement.
   1. Identify and define roles and timeframes for reporting to sponsors and federal and applicable state agencies serious adverse events, serious and continuing non-compliance, unanticipated problems involving risks to subjects or others, or suspension or termination of central IRB approval.
   2. Clearly communicate expectations, including regulatory requirements, sharing of information between the institution and the IRB, and a process for determining potential corrective/remedial actions in the event of non-compliance.
   3. Develop a communication plan for sharing information about the site, the investigators, the sponsor, and the clinical trial between the institution and the IRB.
      1. Identify the plan to evaluate investigator qualifications.^[[1]](#footnote-1)^
      2. Communicate any substantive changes to the institution, its human research program, or the local research context in connection with the clinical trial to the reviewing IRB and vice versa.
   4. Identify a process for responding to participant concerns and grievances, including coordination of communication to subjects.

2. Responsibilities of the **central IRB** **for a multicenter protocol**:

Responsibilities (non protocol-specific):

1. Maintain program for education and training in human subjects research for IRB personnel.
2. Register with FDA and OHRP.
3. Notify institution if accreditation status changes.

Responsibilities (protocol-specific):

1. Ensure clinical trial meets generally accepted ethical standards of human subjects protections and complies with applicable regulations, for example , the Common Rule (45CFR 46), 21 CFR Parts 50, 56, 312, and 812, as well as state and applicable international regulations, such as the European Clinical Trial Directive.
2. Collect, review, and take into account site-specific information provided by the individual sites. This information could include special considerations regarding local populations, or state laws and any restrictions placed on the clinical trial by the institution, such as the need for radiation safety or pharmacy and therapeutic committee review, as well as feasibility of the research or special training requirements.
3. Review and approve the informed consent form and any other research-related documents or media. The process for review and decision could include the following steps:
   1. Provide the investigator/researcher with the sponsor-approved informed consent form. This form should indicate where the institution may add or modify language specific to their site, for example, to the sections on compensation for research-related injury, compensation to subjects, institutional contact information, and costs of participation.
   2. If applicable, review and approve any site-specific modifications.
4. Provide the investigator/researcher with copies of all IRB decisions.
5. Provide the institution with copies of IRB approval documents, IRB rosters, and meeting minutes upon request or in accord with the IRB authorization agreement.
6. Notify the institution promptly in writing of serious or continuing non-compliance or unanticipated problems involving risks to subjects or others.
   1. As appropriate, notify the institution about information from internal and external reports and complaints determined, discovered, or learned by the central IRB in connection with the conduct of a clinical trial by the institution, or in connection with the conduct of the clinical trial by another site if such discovery or determination regarding the other site affects the conduct of the clinical trial at other sites.
7. Notify the institution promptly in writing of any suspension or termination of central IRB approval and of remedial actions required of the institution or its agents by the central IRB.
8. If review is for an institution that conducts federally-funded research, the central IRB must commit to adhere to the requirements of the institution’s federal-wide assurances (FWA(s)).

3. Responsibilities of the **institution** where research is being coordinated or conducted:

Responsibilities (non protocol-specific):

1. Maintain program for education of investigators and research staff and training in human subjects research.
2. Maintain policies and procedures for the conduct of human subjects research as appropriate for the particular institution.
3. Maintain appropriate institution-specific required credentialing of staff.
4. If institution conducts federally-funded research, maintain approved federal-wide assurances FWA(s), including ensuring that the arrangement with the central IRB is documented by a written agreement.
5. Conduct a privacy and security review as required by the Health Insurance Portability and Accountability Act (HIPAA) of 1996 with respect to the mechanisms for permitting the use and disclosure of Protected Health Information (PHI) for clinical trials:
   1. The covered entity (typically the institution) is responsible for the authorization.
   2. The covered entity is responsible for receiving the notice of decedent research.
   3. The covered entity is responsible for the Limited Data Set with Data Use Agreement.
6. Ensure that the investigator/researcher is conducting research and recruiting potential research participants in accordance with IRB-approved protocol, procedures, and documents.

Responsibilities (protocol-specific):

1. Designate the IRB of record for the protocol.
2. Obtain IRB approval of research protocols involving human subjects.^[[2]](#footnote-2)^
3. For PHS-funded research, conduct a conflict of interest (COI) review pursuant to the Public Health Service regulations on Promoting Objectivity in Research, 42 CFR Part 50, Subpart F.
4. Notify the IRB promptly in writing of serious or continuing non-compliance or unanticipated problems involving risks to subjects or others.

4. Responsibilities that **either** the central IRB or the institution could assume, depending on the specific protocol:

1. Evaluate the local context in which the research will be conducted, including consideration of any specific requirements of state or local laws, regulations, policies, or standards. If this responsibility is assumed by the institution, they should inform the central IRB of any relevant requirements or findings from the analysis that would affect conduct of the clinical trial at that institution.
2. If one is requested, provide a waiver of authorization as described under the Health Insurance Portability and Accountability Act (HIPAA) of 1996.

1. This is the responsibility of the IDE/IND holder and the IRB for FDA-related clinical trials. [↑](#footnote-ref-1)
2. This is the responsibility of the Clinical Investigator under FDA-related clinical trials. [↑](#footnote-ref-2)
